# Budget impact analysis of medication reviews by junior pharmacists in polypharmacy patients: a hospital perspective

**DOI:** 10.1007/s11096-026-02190-4

**Published:** 2026-07-17

**Authors:** Toon Hectors, Thirza Kooijman, Marit van Barreveld, Marianne Kuijvenhoven, Patty Teeuwisse, Rik Olde Engberink

**Affiliations:** 1 Division of Systems Pharmacology and Pharmacy, Leiden Academic Centre for Drug Research, Leiden, The Netherlands; 2 Department of Clinical Pharmacy and Toxicology, Leiden University Medical Centre, Leiden, The Netherlands; 3 Department of Clinical Pharmacology and Pharmacy, Amsterdam UMC Location University of Amsterdam, Amsterdam, The Netherlands; 4 Epidemiology and Data Science, Amsterdam UMC Location University of Amsterdam, Amsterdam, The Netherlands; 5 Nephrology, Amsterdam UMC Location University of Amsterdam, Amsterdam, The Netherlands

**Keywords:** Budget impact, Clinical pharmacy, Cost-savings, Medication review, Medication recommendations, Pharmacist

## Abstract

**Introduction:**

Pharmacist-led medication reviews are increasingly recognised for generating cost savings within the hospital. However, no cost-analysis has investigated the budget impact of junior pharmacists conducting medication reviews under supervision of clinical pharmacists, nor included recommendations to start-, increase-, or monitor medications.

**Aim:**

To assess the budget impact of medication reviews by supervised junior pharmacists across hospital wards, explore budget impact differences between drug classes, and identify patient predictors that potentially influence the budget impact.

**Method:**

Data of medication reviews were collected over a 19-month period in patients with polypharmacy (≥ 5 medications) who had at least one additional risk factor. We developed a cost model over a one-year horizon, from the hospital perspective. Intervention costs were derived from measured labour time per medication review and salary of junior- and clinical pharmacists. Cost avoidance from potential preventable medication-related readmissions were based on admission time, admission costs and an estimate of preventable medication-related admissions (4.8%). Costs and savings from medication recommendations were aggregated using drug price per daily defined dose and its predicted days of therapy per year, and clustered by drug class. Scenario analyses tested the robustness of medication recommendation savings to account for effectively implemented and additional monitoring recommendations. Patient predictors of budget impact related to medication recommendations were assessed using multivariable linear regression, including age, gender, eGFR, acute admission, medication count, comorbidity, expensive drugs, and ward type.

**Results:**

We collected 271 medication reviews, including 1,689 medication recommendations. Per-patient budget impact was negative for the intervention (€104 costs), but positive for medication recommendations (€46 savings), and preventable medication-related readmissions (€239 savings). Total estimated savings were €181 per patient, and €688,000 when upscaled hospital-wide. Opiates, beta receptor agonists, corticosteroids, urological drugs, and muscarine receptor antagonists generated 43% of medication recommendation savings. Erythropoietic growth factors, SGLT2 inhibitors, etanercept, etravirine, and calcium/vitamin D formulations generated 70% of medication recommendation costs. No significant associations were found between any of the included patient characteristics and budget impact related to medication recommendations.

**Conclusion:**

Junior pharmacist-led medication reviews provide a modest cost-saving intervention from the hospital perspective, demonstrating savings across clinical populations with polypharmacy.

**Supplementary Information:**

The online version contains supplementary material available at https://doi.org/10.1007/s11096-026-02190-4.

## Impact statements

- From the hospital perspective, junior pharmacists-led medication reviews conducted under clinical pharmacist supervision are associated with modest cost savings, even when cost-increasing recommendations to initiate, intensify, or monitor medication therapy are included.
- The direct budget impact of medication recommendations varies substantially across medications. A limited number of medications accounts for most costs and savings, whereas most recommendations have minimal financial impact.
- The net budget impact of medication recommendations appears largely independent of patient characteristics, suggesting that medication reviews by supervised junior pharmacist are applicable across a broad hospital population with polypharmacy

## Introduction

Medication reviews are structured evaluations of a patient's medication with the aim of optimising its use and improving clinical outcomes. This is particularly relevant in hospital settings where polypharmacy and acute changes in health status are common [1, 2]. Hospitalised patients are at high risk for drug-related problems and consequent harm, with studies reporting that 5.6–30.6% of admissions are medication-related [3, 4]. The optimisation of medication regimens has been associated with reduced drug-related readmissions and improvements in clinical outcomes, quality of life and medication adherence [5–11]. In addition to clinical benefit, recent studies have highlighted pharmacist-led clinical medication reviews as a potentially cost-effective intervention with a positive return-on-investment [12–15]. However, reported benefit-cost ratios vary substantially across settings, ranging from hospital-wide programmes (9.7) to internal medicine wards (1–1.71) and emergency departments (0.47–3.55), with ratios above 1 indicating net savings. While these economic evaluations suggest that pharmacist-led medication reviews may be cost-saving, they do not provide a comprehensive assessment of their overall financial impact. One prior budget impact analysis reported substantial net savings associated with pharmacist-led medication reviews in the hospital [16]. However, all previous reports have largely restricted their analysis to deprescribing recommendations and/or avoided adverse drug events. For a realistic estimation of the costs and savings associated with medication reviews, recommendations to initiate new medications, adjust dosages, or perform additional monitoring should also be incorporated. In addition, the role of students and junior graduates in clinical medication review services is largely absent from the current evidence base, particularly with respect to financial impact. All previous cost-analyses have been limited to models in which (resident) clinical pharmacists conduct medication reviews within clinic settings. Nevertheless, in many countries, pharmacists work in hospitals without formal postgraduate specialisation or registration [17, 18]. To our knowledge, no previous study has evaluated the hospital budget impact of medication reviews conducted by junior pharmacists under the supervision of clinical pharmacists.

### Aim

This study aimed to (1) assess the hospital budget impact of medication reviews by supervised junior pharmacists across hospital wards, including all medication recommendations (both cost-saving and cost-increasing); (2) explore differences in cost impact by drug class; and (3) identify patient predictors that potentially influence the net budget impact.

## Method

### Setting and intervention

In the Amsterdam University Medical Centre, a large tertiary hospital, junior pharmacists routinely conducted medication reviews across various wards under supervision of a clinical pharmacist. Junior pharmacists were defined as early-career pharmacists (≤ 3 years post-graduation) without being in a residency or specialist training track. A clinical pharmacist, as defined by the American College of Clinical Pharmacy (ACCP), works directly with physicians, other health professionals, and patients to ensure that prescribed medication contributes to the best possible health outcomes. In our study, all clinical pharmacists were registered hospital pharmacists that completed a 4-year postgraduate specialisation. Medication reviews could either be initiated by the treating clinician or pharmacist. All reviews were conducted in accordance with a validated format based on the systematic tool to reduce inappropriate prescribing (STRIP) of the Royal Dutch Association for the Advancement of Pharmacy medication review guideline [19, 20].

### Study population

Patients were selected for a medication review based on the following criteria:

1. Polypharmacy (defined as the use of ≥ 5 medications for ≥ 3 months)
2. And at least one of the following risk factors:
3. At least two chronic conditions
4. Impaired renal function (eGFR < 50 ml/min/1.73 m.^2^)
5. Cognitive impairment
6. Increased risk of falling (≥ 1 fall in the previous 12 months)
7. Evidence of decreased medication adherence
8. Suspected medication-related problem

### Data collection

We retrospectively included all medication reviews, performed between September 2023 and April 2025 (19 months). Each medication review represented a single patient. Junior pharmacists collected data on patient demographics, proposed medication recommendations; the implementation of medication recommendations; and specifications of drug related problems (DRP), using a Castor EDC database. Castor is an electronic data capture system in which all study-related data are systematically entered, stored, and managed, allowing structured registration of clinical and medication-related variables for research purposes [21]. For each medication review, supervised junior pharmacists assessed whether the hospital admission was medication-related, using the information documented by the attending physician within the electronic patient record at the time of admission. Medication-related was defined as: caused by one or more medications due to adverse drug reactions, non-adherence, dosing errors or drug–drug interactions. Patients could receive multiple medication recommendations. We predefined 15 frequent recommendations (Table S1), whereas all other recommendations were entered manually. For each recommendation the type of advice (start, stop, alter, evaluate, monitor, other) and its timeline were registered, with one category assigned per recommendation. “Evaluate” relates to the drug therapy in relation to the present disease state. “Monitor” relates to surrogate markers (drug levels, eGFR, blood pressure, etc.). “Other” recommendations included implicit statements (e.g., “consider”, “perhaps”, “if this is the case, then”).

### Budget impact analysis framework and outcomes

The budget impact was estimated over a one-year horizon, from the hospital’s financial perspective, and reported in accordance with the Consolidated Health Economic Evaluation Reporting Standards (CHEERS) 2022 (Table S2) [22]. Cost components included the costs of medication recommendations, the intervention (i.e. labour associated with conducting medication reviews by junior pharmacist under supervision), and potentially preventable medication-related hospital readmissions. Outcomes are presented as mean annual cost savings per patient with 95% confidence intervals and expressed in euros for reference year 2025. For hospital-wide upscaling of the intervention, we assumed 3,800 patients (31% of the average Dutch hospital’s yearly clinical admissions that were longer than 24 h [23, 24]). This was based on the 31–62.5% of patients that are treated with 10 or more medications in the general hospital [25, 26]. Annual costs are, therefore, reported per patient and per 3,800 patients. Analyses were conducted in accordance with the Dutch costing manual for economic evaluations in healthcare [27]. Costs originating from different calendar years were price indexed with general yearly consumer price indices from Statistics Netherlands (Table S3) [28].

### Intervention costs

Across hospital wards, a team of five junior pharmacists measured the time required for each medication review with a stopwatch, as well as the supervision time provided by clinical pharmacists (N = 14). Measurements encompassed the whole process of the intervention, including patient selection, walking time, administration and clinician or patient visits. To convert time into costs, gross monthly salaries were obtained from the 2025 Dutch collective labour agreement for academic hospitals [29]. Fringe benefits and employer’s contributions surcharge of 41% and 35% were added for junior- and clinical pharmacists, respectively [27]. Assuming a full-time equivalent of 36 h per week, the resulting hourly costs were €48.02 for junior pharmacists and €80.28 for clinical pharmacists.

### Budget impact related to medication recommendations

The annual budget impact of medication recommendations was estimated by multiplying the lowest available price per daily defined dose (DDD) by the usual number of therapy days per year (Table S1 provides examples). These were derived from medicijnkosten.nl [30] and the Dutch pharmacotherapeutic compass [31]. For “start” recommendations, the calculation reflects an additional cost; for “stop” recommendations, a cost saving. Cost-savings of “start” or “stop” recommendations were calculated by respectively subtracting or adding yearly price for the corresponding drug. “Alter” and “other” recommendations were individually micro-costed based on the details entered in the Castor database. Costs of “monitor” recommendations were calculated using Amsterdam UMC tariffs of pharmacy-; cardiology-; and clinical chemistry departments [32]. Costs of “evaluate” recommendations were estimated based on the associated drug related problem (DRP): For “undertreatment”, half the annual DDD costs of the corresponding drug was added. For “overtreatment”; “missing indication”; “potential adverse effect”; “contra-indication”; and “drug-drug-interaction”, half of the annual DDD costs were subtracted. Individual micro-costing was also applied for “incorrect dose” or “problem with administration”. Lastly, costs of all medication recommendations were clustered by drug class to explore differences between groups.

### Cost-avoidance related to preventable hospital readmissions

To estimate the potential costs that may be saved due to a reduction in future medication-related readmissions following medication reviews by supervised junior pharmacists, we used data from our cohort and other cohorts. We estimated the number of potential future preventable readmissions and multiplied this by the average cost per readmission. Readmission costs were calculated by multiplying our cohort’s observed mean days of hospital stay (Slicerdicer, Epic electronic health record system) by the unit costs per non-ICU admission day (€665) [27]. From our cohort, we extracted data on the proportion of total admissions that were *medication-related* (10.3%). From previous cohorts, we used data on the proportion of *medication-related* admissions that were *preventable*, ranging from 46.5% to 75% [4, 11]. In our calculations, we used the most conservative 46.5% estimate [11], leading to a proportion of potential *preventable medication-related* readmissions of 4.8% (10.3% × 46.5%). In a sensitivity analysis we applied the most conservative proportion of preventable medication-related admissions that was previously reported in a clinical cohort (2.6% from the HARM study [4]).

### Scenario analyses

Several scenarios were tested to evaluate the potential impact of assumptions on medication recommendation budget impact. In scenario 1 all available medication recommendation costs and savings were included equally. Scenario 2 only included recommendations that were confirmed as implemented, while excluding medication recommendations of which the implementation was not verified. In scenario 3 all “monitor” medication recommendations were excluded, assuming that the cost impact of proposed laboratory measurements may be overestimated.

### Multivariable analysis of budget impact related to medication recommendations

A Gaussian linear regression model was constructed with the net budget impact from medication recommendations per patient and the following candidate predictor variables: age, gender, estimated glomerular filtration rate (eGFR), acute admission, number of clinical- and home medications, Charlson comorbidity index, use of expensive drugs (> 5000 euros per patient per year), and ward type. Predictor variables were chosen a priori, based on clinical relevance and data availability. Patients with missing data for any of the predefined variables (n = 17) were excluded listwise (complete case analysis). We employed heteroscedasticity-robust standard errors (HC3 variant) to ensure reliable inference. Cook's distance diagnostics and a robust regression sensitivity analysis (M-estimation with bisquare weighting) were employed to identify and assess the influence of outliers. All analyses were performed in RStudio (Posit, Boston, MA; version 2025.09.1). Statistical significance was set at α = 0.05.

### Ethics approval

The Medical Ethics Review Committee of the Amsterdam University Medical Centre (UMC) approved the present study (2024.1024).

## Results

### Population characteristics

A total of 271 medication reviews, comprising 1,689 medication recommendations, were included. Admissions concerned an acute setting for 79% of cases and 10.3% of the admissions were related to medication (Table 1). Clinical medication reviews took place on a variety of wards, of which 27% on the internal medicine ward. Mean number of medications at home and in the clinics was 13 and 17, respectively. On average, 7 medication recommendations were given per medication review, of which, 23% advised stopping- and 14% advised starting medications. Recommendations were confirmed as implemented in 48% of cases.

**Table 1:** Characteristics of study population

Characteristic	Mean ± SD or Percentage
Age (years)	69 ± 12
Female sex	42%
Charlson comorbidity score	6 ± 3
eGFR (CKD-EPI, ml/min/1.73 m^2^)	58 ± 33
≥ 90	21%
60–89	28%
30–59	27%
0–29	25%
Hospital admission type	
Planned	21%
Acute	79%
Prescribed drugs at home	13 ± 6
Prescribed drugs in the hospital	17 ± 5
Medication recommendations	7 ± 3
Type of medication recommendation	
Stop	23%
Other	19%
Alter	19%
Evaluate	15%
Start	14%
Monitor	10%
Implementation status	
Followed up	36%
Transmitted	12%
Not implemented	4%
Unknown	48%
Medication-related admission	
Yes	10.3%
No	89.7%
Included wards	
Internal medicine	27%
Cardiology	15%
Otolaryngology	13%
Surgery	12%
Acute admissions unit	11%
Neurology	9%
Vascular surgery	8%
Other	6%

Characteristics were collected from 1,689 medication recommendations to 271 patients in a tertiary hospital, which were selected for a medication review. eGFR = estimated glomerular filtration rate, CKD-EPI = Chronic Kidney Disease Epidemiology Collaboration

### Total budget impact from medication reviews by supervised junior pharmacists across hospital wards

Combining all relevant costs and savings of medication reviews resulted in estimated annual per patient net savings of €181 (95% CI + 63 to + 299, Table 2). Labour time per clinical medication review was 111 (94 to 128) minutes, including 11 (6 to 16) minutes on supervision by a clinical pharmacist. This resulted in intervention costs of €104 (− 119 to − 89) per patient. Per patient medication recommendation costs and savings were €167 (− 380 to + 46) and €213 (+ 152 to + 274), respectively, resulting in a neutral budget impact of €46 (− 53 to + 144). As 10.3% of the admissions in this cohort were medication-related, and previous studies demonstrated that medication reviews can prevent 46.5% of such admissions [4], we assumed that our medication review could prevent a future readmission in 4.8% of our cohort (13 patients). Given that the mean hospital length of stay in this cohort was 7.5 (5.5 to 9.5) days and the costs per admission day were €665, we estimated that our intervention would save €64,735 readmission costs, which corresponds to an average of €239 (+ 175 to + 303) per patient. Hospital-wide upscaling to 3,800 patients could increase savings to €688,000 (240,000 to 1.1 M) per year. In the sensitivity analysis using the most conservative estimate of potentially preventable medication-related readmissions (2.6% instead of 4.8%), the per patient savings were €130 (+ 95 to + 165), resulting in a neutral total budget impact (€72, − 33 to + 177).

**Table 2:** Per patient budget impact from medication reviews

Budget impact	Mean (€)	95% CI (€)
Intervention	− 104	− 119 to − 89
Medication recommendations	+ 46	− 53 to + 144
Preventable medication-related readmissions	+ 239	+ 175 to + 303
Net	+ 181	+ 63 to + 299

Intervention costs were derived from labour time per medication review and gross monthly salaries of Junior- and clinical pharmacists. Medication recommendation budget impact was calculated from price per daily defined dose and estimated days of therapy per year, based on 1,689 individual medication recommendations from 271 junior pharmacist-led medication reviews across hospital wards. Preventable medication-related readmission cost avoidance was estimated from admission time across included wards, admission costs per day and proportion of admissions that was medication-related and preventable. CI = confidence interval

### Scenario analysis of budget impact related to medication recommendations

The estimated budget impact of medication recommendations was assessed under three scenarios. When all medication recommendations were weighted evenly (scenario 1), the net budget impact per patient was neutral €46 (− 53 to + 144). Considering only medication recommendations that were confirmed implemented (scenario 2) resulted in savings of €75 (+ 22 to + 127) per patient. Excluding all “monitor” recommendations (scenario 3) also resulted in a neutral budget impact (€62, − 36 to + 160) per patient.

### Budget impact related to medication recommendations by drug class

The estimated budget impact of medication recommendations varied substantially between drug classes and individual medication recommendations. The five medication groups with the highest estimated cost savings were responsible for 43% of total medication recommendation savings, whereas the five groups associated with the highest costs accounted for 70% of total medication recommendation costs (Table 3). Conversely, the 261 standard deprescribing recommendations (15% of all medication recommendations) which involved stopping or lowering urological drugs, antihypertensives, benzodiazepines, proton pump inhibitors, statins, vitamins, antihistamines, iron supplements, and acetaminophen accounted for only 10% of total medication recommendations savings (Table S1). Moreover, 28% of all medication recommendations were predicted to have negligible budget impact due to minimal differences in price per DDD or in the number of days of therapy per year.

**Table 3:** Budget impact from clinical medication recommendations between drug classes

Rank	Drug (class)	Number issued and % of total medication recommendations	Annual budget impact	Annual savings per patient
Most saving (stop, lower, switch to less expensive drug)				
1	Opioids	40 (2.4%)	€11,260	€42
2	Short or long acting beta receptor agonists	37 (2.2%)	€6,030	€22
3	Corticosteroids (including inhalations and topicals)	34 (2.0%)	€5,264	€19
4	Tamsulosin/solifenacin/mirabegron/dutasteride/tolterodine	38 (2.2%)	€4,624	€17
5	Short or long acting muscarine receptor antagonists	19 (1.1%)	€4,561	€17
Most costly (start, increase, switch to more expensive drug)				
1	Erythropoietic growth factors	4 (0.2%)	€13,260	-€49
2	Sodium glucose transporter 2 inhibitors	33 (2.0%)	€11,771	-€43
3	Etanercept	1 (0.1%)	€7,990	-€29
4	Etravirine	1 (0.1%)	€4,373	-€16
5	Calcium and vitamin D formulations	115 (6.8%)	€4,318	-€16

Annual budget impact was based on 1,689 medication recommendations from 271 junior pharmacist-led medication reviews across hospital wards. Per patient aggregates represent the mean

### Patient predictors of budget impact related to medication recommendations

The multivariable linear regression model, including the patient characteristics age; gender; eGFR; acute admission; number of clinical- and home medications; Charlson comorbidity index; expensive drug use and ward type, explained only 5% of the variance in net budget impact related to medication recommendations from supervised junior pharmacists (R^2^ = 0.052, adjusted R^2^ = -0.079, F = 0.87, *p* = 0.60**, **Table 4). None of the included variables showed a statistically significant association (all *p* > 0.05). Cook’s distance diagnostics revealed no influential outliers (all D < 0.5). Sensitivity analyses indicated unchanged effect directions and coefficient magnitudes of similar order, supporting the stability of the main findings.

**Table 4:** Predictors of budget impact related to medication recommendations

Predictor	Coefficient (€)	95% CI	*p*-value
Age (years)	− 7.2	− 19.2 to 4.7	0.24
Gender (Male vs Female)	− 80.3	− 288.9 to 128.2	0.45
eGFR (CKD-EPI, mL/min/1.73 m^2^)	2.8	− 0.9 to 6.4	0.13
Admission type (acute vs planned)	− 65.7	− 257.4 to 126.0	0.50
Home medications (count)	− 5.5	− 41.3 to 30.2	0.76
Clinical medications (count)	5.7	− 18.7 to 30.1	0.64
Charlson’s comorbidity index	18.2	− 25.5 to 61.9	0.42
Expensive drugs (yes vs no)	− 486.7	− 1676.1 to 702.7	0.42
Ward type	–	–	0.85

Budget impact and predictor variables were derived from 1,689 medication recommendations out of 271 junior pharmacist-led medication reviews across hospital wards. Model was constructed using Gaussian linear regression with heteroscedasticity-robust standard errors (HC3 variant). Expensive drugs are defined as drugs that cost more than 5000 euros yearly per patient

## Discussion

Our findings suggest that junior pharmacist-led clinical medication reviews are cost-saving, or, at minimum, budget neutral within a hospital setting, also when incorporating the negative budget impact from medication recommendations. Future upscaling throughout the hospital population may lead to considerable savings, which is likely not limited to our institutional context. This supports continued investment into medication review strategies.

Our per-patient intervention cost was €104, while medication recommendation and preventable medication-related readmission savings were €46 and €239, respectively, yielding net savings of €181. Although individual cost-components of previous findings may deviate from our estimations, the net budget impact per patient is roughly similar. Wilkes et al. reported €17 in labour costs, €19 in deprescribing savings per patient, and €142 in adverse drug event cost avoidance, leading to €144 of total savings per patient [12]. Kearny et al. estimated €11 in labour costs and €67 in adverse drug event cost avoidance, generating €56 in net savings per patient [16]. Jermini et al. suggested per patient labour costs of €119 and €322 for avoided drug events, resulting in net savings of €203 [13]. Rahman et al. predicted €141 in labour costs and €203 in deprescribing savings, reflecting net savings of €62 per patient [14].

Our analysis is the first to include the full spectrum of recommendations from medication reviews to evaluate their budget impact, whereas prior studies considered only medication reduction or avoided adverse drug events. This may partly clarify discrepancies between our cost-component estimates and those reported previously. Moreover, the previous studies measured an average intervention time of 6, 9, and 113 min per patient, while ours was 111 min. The relatively long duration of a medication review could be attributed to the employment of supervised junior pharmacists, instead of independently working (resident) clinical pharmacists. We also ensured our measurements encompassed the whole intervention process, including patient selection, walking time, administration and clinician or patient visits.

Most reviews included recommendations to discontinue medication but it was also frequently advised to start new medication. When implementation status was taken into account, the estimated cost savings increased. This can be explained by previously reported acceptance patterns for pharmacist-led medication recommendations, where discontinuation advices are accepted more often than initiation recommendations [33].

As compared to a Dutch hospital’s consolidated revenue of €50 million to €2.7 billion [34, 35], our and previously reported savings account for a minor proportion of its complete financial scope. However, we investigated the intervention in its current scale in the Amsterdam UMC, where 380 medication reviews are conducted annually by a team of five junior pharmacists. Upscaling the intervention to 31% of the average Dutch hospital’s annual ≥ 24 h clinical admissions (3,800 patients [23, 24]) could considerably increase savings. Additionally, medication reviews significantly improve clinical outcomes, quality of life and medication adherence [5–11], exceeding their merit beyond that of alternative cost-saving measures.

The budget impact of our medication recommendations suggested considerable differences between drug classes. Notably, individual recommendations concerning highly expensive drugs could substantially influence the total budget impact. Although these results should be interpreted as explorative, prioritisation strategies could capitalise on these discrepancies to enhance cost savings in clinical medication reviews. Our estimates were based on direct cost impact, instead of potential future cost avoidance from adverse drug events. Thus, drug classes that may generate less direct cost impact, but have a high potential for future cost avoidance should still be considered in policies (e.g. antibiotics [36], anticoagulants [37], and hypoglycaemics [38]). Nevertheless, most pivotal drug class targets for clinical medication reviews consist of those that substantially impact both direct costs and cost avoidance [39].

To further target the deployment of medication reviews, we assessed whether patient characteristics were associated with the net costs of medication reviews. Although the model may have been underpowered for economic heterogeneity detection, we did not find any significant predictors of the cost savings, suggesting that junior pharmacist-led medication reviews are applicable across a broad hospital population with polypharmacy.

Our study determined that 10.3% of hospital admissions were medication-related, which aligns with previous reports estimating rates between 5.6% and 30.6% [3, 4]. Given the old age, hyperpolypharmacy, and predominantly acute admission status of our population, this proportion appears plausible [40]. However, as our study was conducted in a tertiary hospital, our results might not exactly represent the general hospital setting or population. For instance, labour costs in primary hospitals may exceed those in academic medical centres, while disease complexity or drug-induced harm may be lower. In the most conservative estimate of potentially preventable medication-related readmissions [4], thereby assuming that our medication review could not prevent a future readmission in 97.4% of our cohort, the net budget impact was neutral.

The specific employment of supervised junior pharmacists might also limit generalisability to other international pharmacist occupations. Analogous positions include staff pharmacist (US); band 6 rotational pharmacist (UK); pharmacien adjoint (France); and non-specialist hospital pharmacist (Japan, Australia). Nevertheless, we demonstrate that pharmacists should not necessarily obtain a specialisation degree to perform medication reviews as a cost-saving intervention in the hospital.

Another considerable limitation is introduced by the single arm design of our study. Consequently, the observed cost savings may partially reflect external influences unrelated to the intervention. For instance, physicians might adopt comparable measures independently, thereby overestimating the effect attributable to the pharmacist intervention. Future randomised controlled trials should incorporate a similar budget impact analysis, taking into account all costs and savings of a medication review. The sustained integration of both patient outcomes and economic benefit in cost-effectiveness studies would elucidate the true merit of the intervention in clinical practice.

We attempted to minimise exaggeration of cost-savings by operating a conservative cost-model using data from this cohort and previous studies. For instance, we assumed that only 46.5% of medication-related admissions were preventable [4], while a meta-analysis reported a 75% risk reduction in medication-related readmissions following pharmacist-led medication reviews [11]. In contrast to other reports, we also comprehensively accounted for the negative financial impact of medication recommendations across different scenarios.

## Conclusion

Our budget impact analysis suggests that medication reviews by supervised junior pharmacists lead to modest cost savings for the hospital. The financial impact depends on drug class and type of recommendation, highlighting opportunities for prioritisation in clinical practice. Further research is needed to confirm cost-effectiveness and generalisability to other hospital settings and patient populations.

## Supplementary Information

Below is the link to the electronic supplementary material.

Supplementary file1 (DOCX 82 KB)

## Data Availability

The datasets generated and/or analysed in this study can be obtained from the corresponding author upon reasonable request.
