# Supplementary material for: Budget impact analysis of medication reviews by junior pharmacists in polypharmacy patients: a hospital perspective
Source: Int J Clin Pharm. 2026 Jul 17;48(5):2073–81. doi: 10.1007/s11096-026-02190-4 (PMC13569556; doi:10.1007/s11096-026-02190-4)
Supplement: Supplementary file 1 [file 11096_2026_2190_MOESM1_ESM.docx]

**Supplementary materials**

**Table S1: Standard medication recommendations from clinical medication reviews**

| **Type of medication**  **recommendation** | **Number issued and % of total medication recommendations** | **Δ of Price per DDD** × **estimated days of therapy per year** | **Based on price per DDD and therapy days of…** | **Yearly savings for included patients (€)** | **Yearly Savings per patient (€)** |
| --- | --- | --- | --- | --- | --- |
| **Start:** calciumcarbonate + cholecalciferol  in case of fracture prevention | 79 (4.7%) | €-0.11 × 365 | Combination tablet | -3,171.85 | -11.70 |
| **Start:** Laxatives in case of opiates | 3 (<0.1%) | €-0.18 × 365 | Macrogol | -197.10 | -0.73 |
| **Discontinue:** alpha blocker/urological spasmolytic due to use >3-6 months | 25 (1.5%) | €0.33 × 365 | Tamsulosin, Solifenacin, Mirabegron, Dutasteride,  tolterodine | 3,047.75 | 11.25 |
| **Discontinue:** antihypertensive medication in case of low blood pressure/risk of falling | 7 (<0.1%) | €0.07 × 365 | Amlodipine, Lisinopril,  Metoprolol, Hydrochlorothiazide, Furosemide, bumetanide | 189.07 | 0.70 |
| **Discontinue:** benzodiazepine due to excessive use/side effects/risk of falling | 28 (1.7%) | €0.07 × 365 | Lorazepam, Temazepam, Oxazepam, zolpidem | 740.95 | 2.73 |
| **Discontinue:** Proton pump inhibitor (by tapering off) due to lack of indication | 56 (3.3%) | €0.08 × 365 | Pantoprazole, Esomeprazole, omeprazole | 1,703.33 | 6.29 |
| **Discontinue:** lipid-lowering medication due to life expectancy/side effects/current lack of indication | 8 (0.5%) | €0.04 × 365 | Simvastatin, Atorvastatin, Rosuvastatin, pravastatin | 102.20 | 0.38 |
| **Discontinue:** vitamin supplementation due to current lack of indication | 14 (0.8%) | €0.05 × 365 | folic acid, vitamin C, thiamine | 229.95 | 0.85 |
| **Dose adjustment:** antihistamine, as needed instead of chronic use | 4 (0.2%) | €0.05 × 365 | Levocetirizine, desloratadine | 73.00 | 0.27 |
| **Dose adjustment:** Direct oral coagulant based on kidney function | 3 (0.2%) | Negligible **Δ** price per DDD | Rivaroxaban, Apixaban,  dabigatran | 0 | 0 |
| **Dose adjustment:** ferrous fumarate, to 200 mg 2-3 times per week | 8 (0.5%) | €0.11 × 130 | from 7 to 2.5 times a week | 327.04 | 1.21 |
| **Dose adjustment:** acetaminophen, reduce due to chronic use | 42 (2.5%) | €0.06 × 365 | 2 tablets per day of 500mg less | 919.80 | 3.39 |
| **Dose adjustment:** proton pump inhibitor, reduce to stomach protection dose | 66 (3.9%) | Negligible **Δ** price per DDD | Same price between products | 0 | 0 |
| **Dose adjustment:** nadroparin, adjustment based on body weight | 8 (0.5%) | €-1.94 × 21 | Mostly 2850IU 1dd1 to 5700IU 1dd1, Mean therapy days is 21 days a year for Amsterdam UMC (2024) | -325.92 | -1.20 |
| **Dose adjustment:** thiamine, change to 50 mg twice daily | 7 (0.4%) | €0.06 × 365 | Four times daily to twice daily | 127.75 | 0.47 |

Annual mean per patient savings were based on 1,689 medication recommendations from 271 junior pharmacist-led medication reviews across hospital wards

**Table S2: Consolidated Health Economic Evaluation Reporting Standards 2022 (CHEERS 2022) checklist**

Note: NA = not applicable

| **Topic** | **No.** | **Item** | **Location where item is reported** |
| --- | --- | --- | --- |
| **Title** |  |  |  |
|  | 1 | Identify the study as an economic evaluation and specify the interventions being compared. | Title, Page 1 |
| **Abstract** |  |  |  |
|  | 2 | Provide a structured summary that highlights context, key methods, results, and alternative analyses. | Abstract, Page 2 |
| **Introduction** |  |  |  |
| **Background and objectives** | 3 | Give the context for the study, the study question, and its practical relevance for decision making in policy or practice. | Introduction, page 3-4 |
| **Methods** |  |  |  |
| **Health economic analysis plan** | 4 | Indicate whether a health economic analysis plan was developed and where available. | NA for budget impact analysis |
| **Study population** | 5 | Describe characteristics of the study population (such as age range, demographics, socioeconomic, or clinical characteristics). | Method, study population |
| **Setting and location** | 6 | Provide relevant contextual information that may influence findings. | Method, setting and intervention |
| **Comparators** | 7 | Describe the interventions or strategies being compared and why chosen. | Method, setting and intervention |
| **Perspective** | 8 | State the perspective(s) adopted by the study and why chosen. | Method, Budget impact analysis framework and outcomes |
| **Time horizon** | 9 | State the time horizon for the study and why appropriate. | Method, Budget impact analysis framework and outcomes |
| **Discount rate** | 10 | Report the discount rate(s) and reason chosen. | NA for a study horizon that does not exceed one year |
| **Selection of outcomes** | 11 | Describe what outcomes were used as the measure(s) of benefit(s) and harm(s). | NA for budget impact analysis (no clinical outcomes measured) |
| **Measurement of outcomes** | 12 | Describe how outcomes used to capture benefit(s) and harm(s) were measured. | NA for budget impact analysis (no clinical outcomes measured) |
| **Valuation of outcomes** | 13 | Describe the population and methods used to measure and value outcomes. | NA for budget impact analysis (no clinical outcomes measured) |
| **Measurement and valuation of resources and costs** | 14 | Describe how costs were valued. | Method, intervention costs; Cost-avoidance related to preventable hospital readmissions; Budget impact related to medication recommendations |
| **Currency, price date, and conversion** | 15 | Report the dates of the estimated resource quantities and unit costs, plus the currency and year of conversion. | Method, Budget impact analysis framework and outcomes, supplementary materials |
| **Rationale and description of model** | 16 | If modelling is used, describe in detail and why used. Report if the model is publicly available and where it can be accessed. | Method, Cost-avoidance related to preventable hospital readmissions; Budget impact related to medication recommendations; Multivariable analysis of budget impact related to medication |
| **Analytics and assumptions** | 17 | Describe any methods for analysing or statistically transforming data, any extrapolation methods, and approaches for validating any model used. | Method, Cost-avoidance related to preventable hospital readmissions; Multivariable analysis of budget impact related to medication |
| **Characterising heterogeneity** | 18 | Describe any methods used for estimating how the results of the study vary for subgroups. | Method, Budget impact related to medication recommendations (last sentence) |
| **Characterising distributional effects** | 19 | Describe how impacts are distributed across different individuals or adjustments made to reflect priority populations. | Method, Multivariable analysis of budget impact related to medication recommendations |
| **Characterising uncertainty** | 20 | Describe methods to characterise any sources of uncertainty in the analysis. | Method, Cost-avoidance related to preventable hospital readmissions (sensitivity analhysis); scenario analysis; Multivariable analysis of budget impact related to medication recommendations (sensitivity analysis) |
| **Approach to engagement with patients and others affected by the study** | 21 | Describe any approaches to engage patients or service recipients, the general public, communities, or stakeholders (such as clinicians or payers) in the design of the study. | Not reported |
| **Results** |  |  |  |
| **Study parameters** | 22 | Report all analytic inputs (such as values, ranges, references) including uncertainty or distributional assumptions. | Results, Total budget impact from junior pharmacist-led medication reviews across hospital wards; Scenario analysis of budget impact related to medication recommendations, Predictors of budget impact related to medication recommendations |
| **Summary of main results** | 23 | Report the mean values for the main categories of costs and outcomes of interest and summarise them in the most appropriate overall measure. | Results, Total budget impact from junior pharmacist-led medication reviews across hospital wards; Table 2 |
| **Effect of uncertainty** | 24 | Describe how uncertainty about analytic judgments, inputs, or projections affect findings. Report the effect of choice of discount rate and time horizon, if applicable. | Results, Total budget impact from junior pharmacist-led medication reviews across hospital wards (last sentence); Scenario analysis of budget impact related to medication recommendations |
| **Effect of engagement with patients and others affected by the study** | 25 | Report on any difference patient/service recipient, general public, community, or stakeholder involvement made to the approach or findings of the study | Not reported |
| **Discussion** |  |  |  |
| **Study findings, limitations, generalisability, and current knowledge** | 26 | Report key findings, limitations, ethical or equity considerations not captured, and how these could affect patients, policy, or practice. | Discussion |
| **Other relevant information** |  |  |  |
| **Source of funding** | 27 | Describe how the study was funded and any role of the funder in the identification, design, conduct, and reporting of the analysis | End of manuscript |
| **Conflicts of interest** | 28 | Report authors conflicts of interest according to journal or International Committee of Medical Journal Editors requirements. | End of manuscript |

**Table S3: Reference values for cost calculations**

| **Category** | **Item** | **Reference value (before indexing)** | **Source (year)** |
| --- | --- | --- | --- |
| Intervention costs | Monthly gross salary junior pharmacist (scale 10-M-7) | €5,313 | [1](2025) |
|  | Monthly gross salary clinical pharmacist (scale 16-5) | €9,277 |  |
|  | Fringe benefits and employer’s contributions surcharge for junior pharmacists | 41% | [2](2024) |
|  | Fringe benefits and employer’s contributions surcharge for clinical pharmacists | 35% |  |
| Medication budget impact | Price per daily defined dose, usual days of therapy per year | See sources for info per individual drug | [3][4](2025) |
| “Monitor” recommendations costs | Sodium, potassium, eGFR (creatinine), calcium, phosphate, cholesterol, glucose, uric acid, haemoglobin, mean corpuscular volume, ASAT, ALAT, thrombocyte count, leukocyte count | €2.50 | [5] (2023) |
|  | Magnesium | €4 |  |
|  | INR, lithium | €6 |  |
|  | CD4 count, folic acid | €8 |  |
|  | HBA1C, prolactin, vitamin B12 | €9 |  |
|  | Thyrotropin | €11 |  |
|  | Parathyroid hormone, cystatin C | €12 |  |
|  | Albumin, digoxin | €14 |  |
|  | Hepatitis-B surface antigen | €25 |  |
|  | Vitamin D | €28 |  |
|  | Ammonia | €29 |  |
|  | H. Pylori | €30 |  |
|  | Thiamin | €34 |  |
|  | Amiodarone, clozapine, posaconazole, valproic acid, Lamotrigine | €49 |  |
|  | Anti-Xa | €52 |  |
|  | Mycophenolic acid, everolimus, sirolimus, tacrolimus, 6-thioguanin | €69 |  |
|  | Electrocardiogram | €104 |  |
|  | CYP450 genotype | €114 |  |
|  | Bone density (DEXA scan) | €186 |  |
|  | Echocardiogram | €228 |  |
| Cost-avoidance related to preventable hospital admissions | Non-ICU admission day (including labour costs) | €644 | [2](2024) |
|  | Most conservative proportion of medication related hospital admissions | 5.6% | [6](2008) |
|  | Proportion of medication-related hospital admissions that are avoidable | 46.5% |  |
| Per patient comparison with previously reported cost components | Wilkes (labour, adverse drug event avoidance, deprescribing), per 558 patients | €7,612; €64,911; €8,660 | [7](2021) |
|  | Kearney (labour, adverse drug events), per patient | €9.40, €55.28 | [8](2018) |
|  | Jermini (labour, adverse drug event avoidance), per 973 patients | €112,408; €304,170 | [9](2024) |
|  | Rahman (labour, deprescribing), per patient | €138, €199 | [10](2024) |
| Intervention upscaling | Yearly clinical admissions (excluding sub-24-hour admissions) in the Netherlands | 1,396,000 | [11](2023) |
|  | Hospital facilities (excluding outpatient clinics) in the Netherlands | 113 | [12](2025) |
|  | Most conservative proportion of patients that use 10 or more medications | 31% | [13](2024) |

Costs originating from before 2025 were price-indexed with general yearly consumer price indices from Statistics Netherlands [14].

**References**

1. Nederlandse Federatie van Universitaire Medische Centra. CAO Universitair Medische Centra 2024–2025 [Collective Agreement University Medical Centres 2024–2025]. Amsterdam: NFU; 2024.
2. Hakkaart-Van Roijen L, Peeters S, Kanters T. Kostenhandleiding voor economische evaluaties in de gezondheidszorg: methodologie en referentieprijzen [Dutch manual for costing in economic evaluations: methodology and reference prices]. In opdracht van Zorginstituut Nederland; 2024.
3. Medicijnkosten.nl. [medicationcosts.nl] https://www.medicijnkosten.nl/. Accessed 26.06.2025.
4. Farmacotherapeutisch Kompas. Farmacotherapeutisch Kompas. Amstelveen: Zorginstituut Nederland; 2016.
5. Amsterdam UMC. ODV-tarieven Amsterdam UMC 2023 [ODV rates Amsterdam UMC 2023]. Amsterdam: Amsterdam UMC; 2023.
6. Leendertse AJ, Egberts ACG, Stoker LJ, et al. Frequency of and risk factors for preventable medication-related hospital admissions in the Netherlands. Arch Intern Med. 2008;168:1890–6. https://doi.org/10.1001/archinternmed.2008.3
7. Wilkes S, Zaal RJ, Abdulla A, et al. A cost–benefit analysis of hospital-wide medication reviews: a period prevalence study. Int J Clin Pharm. 2022;44:138–45. https://doi.org/10.1007/s11096-021-01323-1
8. Kearney A, Walsh EK, Kirby A, et al. A budget impact analysis of a clinical medication review of patients in an Irish university teaching hospital. Glob Reg Health Technol Assess. 2018;5. https://doi.org/10.1177/2284240318807726
9. Jermini M, Fonzo-Christe C, Blondon K, et al. Financial impact of medication reviews by clinical pharmacists to reduce in-hospital adverse drug events: a return-on-investment analysis. Int J Clin Pharm. 2024;46:496–505. https://doi.org/10.1007/s11096-023-01683-w
10. Rahman RN, Polinder S, Nikolik B, et al. Medication reviews by emergency department pharmacists in patients hospitalised for an adverse drug event: a cost study. BMC Health Serv Res. 2024;24:1–9. https://doi.org/10.1186/s12913-024-11346-9
11. Rijksinstituut voor Volksgezondheid en Milieu. Ziekenhuiszorg | Gebruik | Volksgezondheid en Zorg [Hospital care | Use]. https://www.vzinfo.nl/ziekenhuiszorg/gebruik. Accessed 31.01.2026.
12. Rijksinstituut voor Volksgezondheid en Milieu. Ziekenhuiszorg | Aanbod | Instellingen | Volksgezondheid en Zorg [Hospital care | Supply | Institutions]. https://www.vzinfo.nl/ziekenhuiszorg/aanbod/instellingen. Accessed 31.01.2026.
13. Alqurain AA, Albaharnah M, Al Zayer S, et al. The prevalence of polypharmacy and hyper-polypharmacy among middle-aged vs. older patients in Saudi Arabia: a cross-sectional study. Front Pharmacol. 2024;15:1357171. https://doi.org/10.3389/fphar.2024.1357171
14. Centraal Bureau voor de Statistiek. Consumentenprijzen; prijsindex 2015=100 [Consumer prices; price index 2015=100]. https://www.cbs.nl/nl-nl/cijfers/detail/83131NED. Accessed 28.01.2026
